# Solid-State Reactions for the Storage of Thermal Energy

**DOI:** 10.3390/nano9020226

**Published:** 2019-02-07

**Authors:** Stefania Doppiu, Jean-Luc Dauvergne, Elena Palomo del Barrio

**Affiliations:** 1 Centro de Investigación Cooperativa de Energías Alternativas, CIC energiGUNE, 01510 Vitoria-Gasteiz, Spain; jldauvergne@cicenergigune.com (J.-L.D.); epalomo@cicenergigune.com (E.P.d.B.); 2 Ikerbasque, Basque Foundation for Science, 48013 Bilbao, Spain

**Keywords:** solid state reactions, thermal energy storage, nanocrystalline materials, ball milling

## Abstract

In this paper, the use of solid-state reactions for the storing of thermal energy at high temperature is proposed. The candidate reactions are eutectoid- and peritectoid-type transitions where all the components (reactants and reaction products) are in the solid state. To the best of our knowledge, these classes of reactions have not been considered so far for application in thermal energy storage. This study includes the theoretical investigation, based on the Calphad method, of binary metals and salts systems that allowed to determine the thermodynamic properties of interest such as the enthalpy, the free energy, the temperature of transition, the volume expansion and the heat capacity, giving guidelines for the selection of the most promising materials in view of their use for thermal energy storage applications. The theoretical investigation carried out allowed the selection of several promising candidates, in a wide range of temperatures (300–800 °C). Moreover, the preliminary experimental study and results of the binary Mn-Ni metallic system are reported. This system showed a complex reacting behavior with several discrepancies between the theoretical phase diagram and the experimental results regarding the type of reaction, the transition temperatures and enthalpies and the final products. The discrepancies observed could be due both to the synthesis method applied and to the high sensitivity of the material leading to partial or total oxidation upon heating even if in presence of small amount of oxygen (at the ppm level).

## 1. Introduction

The scope of this investigation is the development of performance materials with high energy density, reversibility, long cycle life, compact, low cost and with the potential to build “simple” thermal energy storage systems. This field of research is one of the priorities for i) helping the penetration and dispatchability of renewable energies; ii) contributing to create a low-carbon society for environmental protection; and iii) increasing energy efficiency and decreasing energy demand as targeted in the main road maps [1,2,3] related to energy policy and environment developed in recent years. In this context, the development of TES materials will play a major role, for example, in helping to the re-utilization of wasted heat (e.g., in industrial processes) and in guaranteeing non-stop energy production, e.g., in solar energy power plants [4,5].

As is already well known, thermal energy can be stored using different processes: sensible, latent and thermochemical storage [5,6,7,8,9]. The energy capacity in these processes increases progressively from sensible to thermochemical processes. Unfortunately, this is accompanied by an increase of the complexity of the TES system that has to be developed, implying a substantial increase of the costs. For example, in the case of gas–solid reactions, the TES system should be composed of two reactors to keep the reactants separated (gas and solid) up to when the discharging process is needed (putting in contact the reactants to promote the exothermic reaction to recover the energy). This solution is technologically much more complex than the case of sensible storage, where the storage material, solid or liquid, is placed in a unique reactor. As a consequence, thermochemical storage is still at the prototyping/demonstration level and implies, so far, high investment costs. Latent heat storage (using solid–liquid or solid–solid phase transitions) is a more accessible technology nowadays at the demonstration level. Sensible storage is a mature technology already commercialized in many applications.

The main idea of this study is the use of solid-state chemical reactions as materials for thermal energy storage at high temperature. In particular, the focus is given to euteuctoid and peritectoid reactions [10] occurring in binary metals and salt-based systems. The goal was the identification of reactions fulfilling the requirements needed to be used as TES materials (high storage capacity, good thermal conductivity, mechanical and chemical stability, complete reversibility in charging/discharging cycles, affordable cost, etc.) and to obtain the experimental proof of their feasibility and reversibility. 

These types of reactions have not been considered so far for the application addressed in this paper. The great advantages and novelties that they are potentially expected to bring in the TES field are as follows:

- Reactants and reaction products in the solid state can make it possible to conceive a TES system with the characteristic of simplicity the sensible storage system (only one reactor).
- Possible direct contact of the reacting material with the heat transfer fluid (allowing minimizing the use of expensive heat exchanger).
- Potential high energy density materials, allowing compact and low-cost systems.

These reactions offer many advantages, but can also present drawbacks, such as problems connected to the atomic diffusion in the solid-state (slow reaction kinetics) and the poor heat transfer rate in the solid-state especially when salt mixtures are taken into account. 

The work included the selection of the most promising materials by a deep analysis of the existing databases of binary metals and salt systems [11], determining all the theoretical thermodynamic properties needed for the evaluation of the TES performances. As a result of the selection process, the Mn-Ni system was chosen for experimental investigation and feasibility study. 

## 2. Materials and Methods 

### 2.1. Materials Selection 

The search for eutectoid and peritectoid reactions with suitable reaction temperatures was based on available phase diagrams for multi-component systems (ASM International, Scientific Group Thermodata Europe, ThemoTech Inc, etc. (GU2 7YG, Guildford, United Kingdom) and focused on metallic and salt binary systems. The theoretical performances were evaluated by using the CALPHAD (CALculation of PHAse Diagram) method. Regular and sub-regular solution models were used to obtain the Gibbs energy functions of various solution phases. The excess Gibbs energy of each phase was represented by the Redlich-Kister formalism, with binary interaction parameters following the form of power series [12,13,14]. These parameters were optimized by using the optimization module of FactSage7.0 software (7.0, GTT-Technologies, Herzogenrath, Germany) [15]. In particular, the selection was performed by using the set of evaluated and optimized thermodynamic databases for inorganic systems, such as light metal, alloy, molten salt, oxide. All the key thermodynamic properties (e.g., enthalpy of reaction, specific heats, densities, volume change during the charge/discharge process) of identified eutectoids and peritectoids were obtained assuming equilibrium conditions. 

### 2.2. Nanocrystalline Materials Production

Two compositions in the Mn-Ni phase diagram were selected for experimental study: the peritectoid Mn_75_-Ni_25_ and the eutectoid Mn_52_-Ni_48_ (molar ratio). Mn and Ni powder were supplied by Alfa Aesar with purities of 99.3% and 99.8%, respectively. To avoid air contamination, the handling and sampling were carried out under controlled atmosphere in an Argon glove box (Brown) with levels of oxygen and humidity lower than 0.1 ppm. 

To maximize the reactivity in the solid state, the materials were subjected to mechanochemical treatment (Ball milling) to achieve powders with a controlled degree of nanocrystallization. The mechanical milling was used only for the synthesis of nanostructured powder. The goal here was not to promote the reaction by ball milling but the preparation of highly reactive materials (high amount of defects, high specific surface area, and high contact area) and activate the reaction subsequently by thermal treatment. 

For this purpose, a Spex mixer mill (875 RPM), using stainless steel vials and balls, was used. Two different milling procedures for the preparation of nanocrystalline materials were applied:

(1) Two-step synthesis: ball milling of separated Mn and Ni using 2 balls of 8 mm diameter. A milling time of 4 h was applied to the pure materials with a ball-to-powder mass ratio (BPR) of 1.6. To obtain a good intermixing between Mn and Ni, the materials were placed in the ball milling reactor in the right molar ratio (Mn_75_-Ni_25_ and Mn_52_-Ni_48_) and subjected to mechanical treatment under mild conditions (for 15 min using 3 balls of 3 mm diameter).
(2) One-step synthesis: ball milling of Mn and Ni directly in the right molar ratio (75/25, 52/48) with 2 balls of 8 mm diameter. A milling time of 4 h was applied with a BPR of 1.6.

### 2.3. Structural Analysis

The structural analysis of the materials was performed by X-Ray diffraction analysis using a Bruker D8 Discover equipped with a LYNXEYE XE detector with monochromatic Cu Kα1 radiation of λ = 1.54056 Å. Patterns were recorded in a 2θ angular range 10–120° with a step size of 0.02° and a step time of 1.5 s. The measurements were performed at room temperature. The structural evolution upon heating was studied by in situ XRD measurements by using a Bruker Advance D8 instrument with cobalt radiation (λCoα1 = 1.78886 Å/λCoα2 = 1.79277 Å). The equipment operated in Brag-Brentano theta-theta geometry, with an operating power of 30 kV and 50 mA. The samples were placed in a nickel-coated high-vacuum chamber designed for the use in the range from room temperature up to 1200 °C (HTK 1200N) under a high vacuum, inert and reactive atmosphere. The sample was mounted on an alumina sample holder avoiding any contact with the wall of the chamber and in contact with the temperature sensor. 

Information about the phases formed and their relative percentages, the crystallite sizes and the microstrain level were obtained from the X-ray patterns by using a full profile fitting procedure [16] based on the Rietveld method [17].

The morphology of the material was studied by Scanning Electron Microscopy (SEM, ) using a Quanta 200 FEG scanning electron microscope (FEI Company, Hillsboro, OR, USA) operated in high-vacuum mode at 30 kV and with a back-scattered electron detector (BSED). In addition, energy-dispersive X-ray spectroscopy (EDX) analyses were carried out in order to obtain chemical composition maps.

### 2.4. Reactivity and Thermodynamic Characterization

The reactivity of the materials was tested by Differential Scanning Calorimetry (DSC) technique using a Thermal Analysis Q2000 model. These techniques allowed the determination of the reaction temperatures and the reaction enthalpies. For all the measurements the heating rate was 5 K/min with three or twenty cycles between 450 and 660 °C including isothermal steps of 30 min between subsequent heating and cooling processes. The structural changes of the materials after DSC experiments were determined by XRD analysis. 

## 3. Results and Discussion

### 3.1. Materials Selection Results

More than 200 binary phase diagrams (metals and salts) were analyzed using available databases and the FactSage7.0 software (7.0, GTT-Technologies, Herzogenrath, Germany) in the temperature range of 300–1000 °C. The criteria of selection were based on availability of the materials, no toxicity and relatively low cost. The modelling made it possible to identify all the transitions of interest in the systems studied (eutectoids, peritectoids, peritectics, eutectics, etc.), together with the associated energy densities and main thermodynamic and thermophysical parameters. In this study, all the systems with eutectoid and peritectoid transitions with theoretical volumetric energy densities lower than 100 kWh/m^3^ were discarded. The results of the theoretical modelling allowed the selection of several potential candidates, in a wide range of temperatures, shown in Table 1, that could be used for further experimental investigation. 

**Figure 1:**
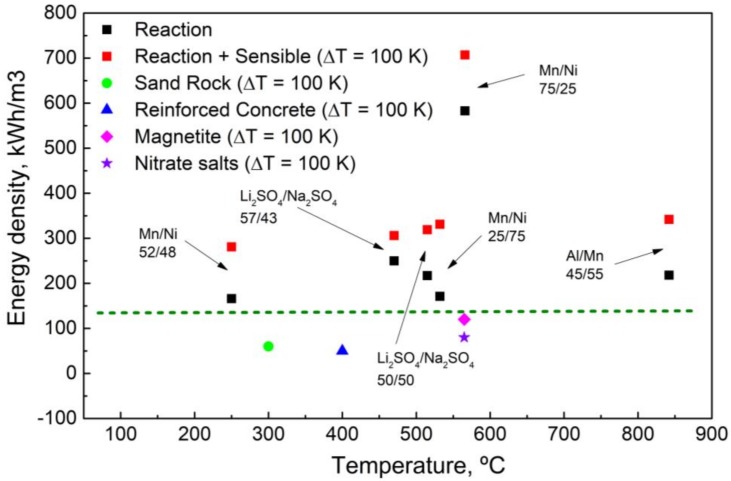
Theoretical volumetric energy densities of some selected solid-state reactions (black squares). The energy corresponding to a ΔT of 100 K (red squares), together with the values relevant to the best sensible storage materials (ΔT = 100 K), is also pictured.

Another aspect considered in this study was how to compare these reactions with other types of thermal energy storage processes (sensible, latent or thermochemical), thinking about their possible integration into a real application. To that end, several aspects have to be considered: (i) we deal with chemical reactions where all the components, reagents and products, are in the solid state; (ii) the reaction mechanism is governed by the atomic diffusion in the solid state; and (iii) the reaction occurs at a well-defined constant temperature. Considering the solid-state nature of these materials, they can be assimilated to a sensible storage material with the difference that at a certain temperature an extra contribution to the sensible heat is given by the enthalpy of the reaction. As a result, depending on the reaction and its energy and on the thermophysical properties, such as the Cp, for the reactions proposed in this paper a higher overall energy density is expected. In Figure 1, the energy density obtained by the sum of the sensible heat contribution and the reaction enthalpy, considering a range of temperature of 100 K around the reaction temperature, is compared to the best sensible storage materials. 

This allows achieving, for almost all the systems considered, theoretical energy densities between 250–350 kWh/m^3^. These values are higher than the sensible storage materials considered nowadays. For example, the magnetite, the best material identified so far, presents an energy density of 120 kWh/m^3^ for a ΔT = 100 K. These results are very promising and confirm the great theoretical potential of these reactions. It is noteworthy that the Mn_75_-Ni_25_ shows theoretical energy densities considerably higher compared to the other systems (583 kWh/m^3^ for the reaction and 707 kWh/m^3^ when a ΔT of 100 K is considered). Due to these results, the Mn-Ni system was the first choice for the experimental investigation. 

**Figure 2:**
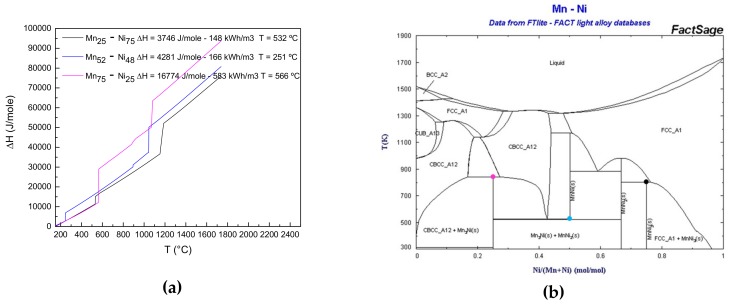
(**a**) Theoretical energy densities of the solid-state reactions corresponding to the compositions Mn_75_-Ni_25_, Mn_52_-Ni_48_ and Mn_25_-Ni_75_. The relative volumetric energy densities are also reported in the Figure. In (**b**), the Mn-Ni phase diagram is shown, with the corresponding transition highlighted (colored dots).

To determine univocally the composition with the highest energy density, for each system studied and each transition of interest, four compositions around the theoretical one were analyzed (two before and two after). In particular, the Mn-Ni system presents three solid-state reactions (both eutectoid and peritectoid) below 600 °C at the compositions Mn_75_-Ni_25_, Mn_52_-Ni_48_ and Mn_25_-Ni_75_ with promising theoretical energy densities as shown in Figure 2. 

### 3.2. Synthesis of Mn_75_-Ni_25_ and Mn_52_-Ni_48_ Nanocrystalline Materials

**Figure 3:**
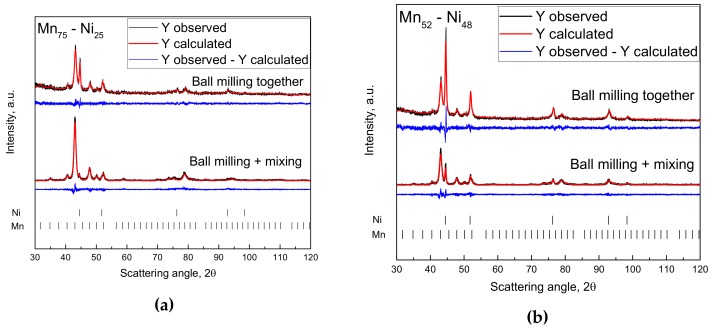
X-ray diffraction results of the samples (**a**) Mn_75_-Ni_25_ and (**b**) Mn_52_-Ni_48_ prepared by one-step or two-step synthesis. The results of the fitting procedure using the Rietveld method are also reported. Black line: experimental pattern. Red line: theoretical pattern. Blue line: difference between experimental and theoretical patterns.

The reactions studied in this paper are governed by the diffusion in the solid state. As a consequence, it is imperative to find synthesis routes in order to maximize the atomic diffusion by decreasing the atomic diffusion path length (small grain sizes as well as small particle sizes), introducing structural defects (dislocation, grain boundaries step, kink and corner atoms, etc.) and promoting high intermixing degree to guarantee the maximum contact between the reagents (high specific surface area). It is well known that a powerful tool for achieving these results is given by mechanochemical techniques [18]. The two procedures applied for the synthesis of nanocrystalline Mn_75_-Ni_25_ and Mn_52_-Ni_48_ led to the formation of samples with different microstructures and similar degree of nanocrystallization (grain sizes around 15 nm) and, as wanted, with no evidence of the formation of the intermetallic Mn_3_Ni and MnNi predicted in the phase diagram. The two compositions studied showed different behavior depending on the milling treatment applied. In Figure 3, the XRD patterns for the four samples are reported together with the results of the fitting procedure using the Rietveld method. Only the Mn and Ni reflections are detected in the XRD patterns.

The two-step synthesis caused, for all Mn_75_-Ni_25_ samples tested in this investigation, the gradual disappearance of the diffraction peaks corresponding to Nickel (PDF number: 01-071-3740 4-850) (see Figure 3a pattern below). Surprisingly, the gradual disappearance of Ni reflections is not accompanied by a substantial variation of the cell parameters of Mn (PDF number: 01-089-2105 32-637), as a consequence of the substitution of Mn atoms by Ni in the Mn net with the formation of a Mn_1−x_Ni_x_ solid solution (see Table 1). The solubilization of Ni into Mn should lead to the decrease of the volume of the primitive cell due to the smaller size of Ni compared to Mn (reference value for Mn being the sample milled 4 h, see Table 2). 

**Figure 4:**
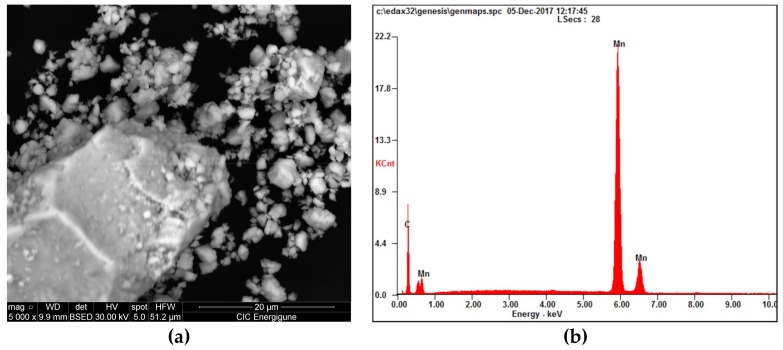
SEM micrograph (**a**) and EDX analysis (**b**) of the samples Mn_75_-Ni_25_ (two-step synthesis).

The sample prepared by one-step synthesis (where the Ni is clearly visible after 4 h of milling, Figure 3a pattern above) shows the expected behavior (decreasing of the Mn cell parameter), while the sample prepared by two-step synthesis (where the Ni is hardly visible after 4 h of milling, Figure 3a pattern below) shows an opposite trend, with slightly higher cell parameters compared to the Mn milled for 4 h. Further studies were then carried out, by applying mechanical milling progressively higher (30 min, 1 h, 2 h, 4 h and 8 h), in order to clarify this behavior and correlate the disappearing of Ni reflection with the structural modification of Mn. For higher milling times (8 h and 16 h) no Ni was detected in the mixture after mixing (XRD). In Figure 4, the SEM micrograph with the corresponding EDX analysis of the sample milled for 8 h are reported, confirming the results obtained by XRD investigation. 

For this sample, the particles size distribution was determined by following two approaches a) using a particles size analyzer (Master sizer 3000, Malvern), and b) using the software ImageJ (version 2.0, an open source Java-based software) [19,20]. Following the two approaches, similar results were obtained within media of around 4 μm (SEM/ImageJ) and around 6 μm (particle size analyzer). The difference between the crystallite sizes and the particle sizes is not surprising, due to the phenomena occurring during milling that, in the case of metals, can promote the cold welding of the particles with a consequent increase of their sizes without decreasing the overall reactivity of the material if the crystallite sizes remain small. The behavior under milling and the final structure obtained, depending on the conditions applied, are now subject to deep study due to the very different reacting behavior observed for the different samples, as will be explained later in the text. 

### 3.4. Reactivity upon Heating

**Figure 5:**
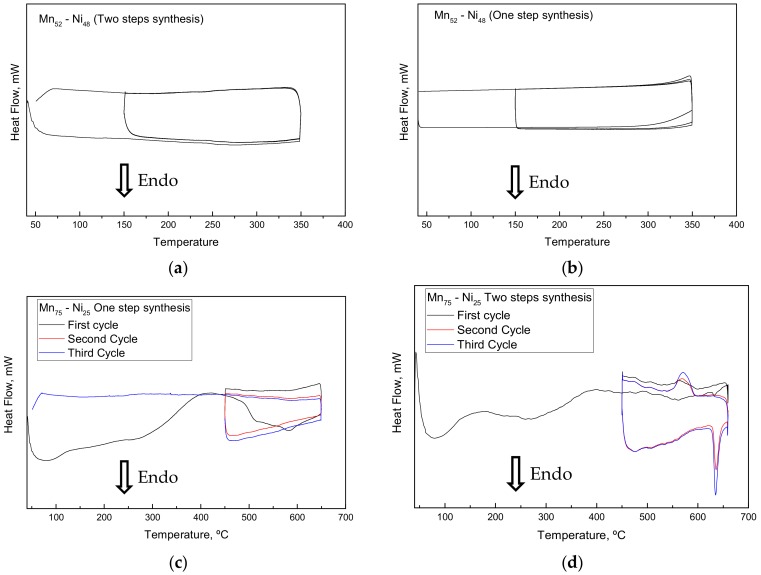
DSC results of the samples: (**a**) Mn_52_-Ni_48_ prepared by two-step synthesis, (**b**) Mn_52_-Ni_48_ prepared by one-step synthesis, (**c**) Mn_75_-Ni_25_ prepared by two-step synthesis, and (**d**) Mn_75_-Ni_25_ prepared by one-step synthesis.

To study the reactivity upon heating and cooling, all the samples synthesized were studied by differential scanning calorimetry and simultaneous thermal analysis techniques. The goal was to determine the reactivity, to quantify the energy relative to the solid-state reaction (peritectoid, eutectoid), and to perform preliminary study on the reversibility by cycling test. The results of the DSC tests are shown in Figure 5.

Regarding the DSC results several aspects can be highlighted: 

There was a discrepancy between the theoretical phase diagram (see inset Figure 1) and the experimental results for the composition Mn_52_-Ni_48_, where no reactivity was detected in that range of composition between room temperature and 350 °C (eutectoid reaction expected at 251 °C). This result was confirmed by further experiments carried out under the same experimental conditions. 

The reacting behavior of the composition Mn_75_-Ni_25_ is strongly influenced by the experimental conditions applied for the synthesis of the material (no reactivity in case of one-step synthesis).

Following the theoretical phase diagram at the composition Mn_75_-Ni_25_, the reaction between Mn and Ni should lead to the formation of the intermetallic Mn_3_Ni. This reaction was expected to be exothermic, while an endothermal event was observed in the experimental results at higher temperature than the predicted one (630 °C instead of 566 °C). Moreover, the XRD patterns performed after DSC measurements reveal, for most of the samples, only the presence of Mn, Mn_(1−x)_Ni_x_O and traces of Ni. The detection of the formation of a small amount of one new “unknown phase” was possible only after many experiments, where the extensive oxidation of the sample during the DSC experiment was limited. 

**Figure 6:**
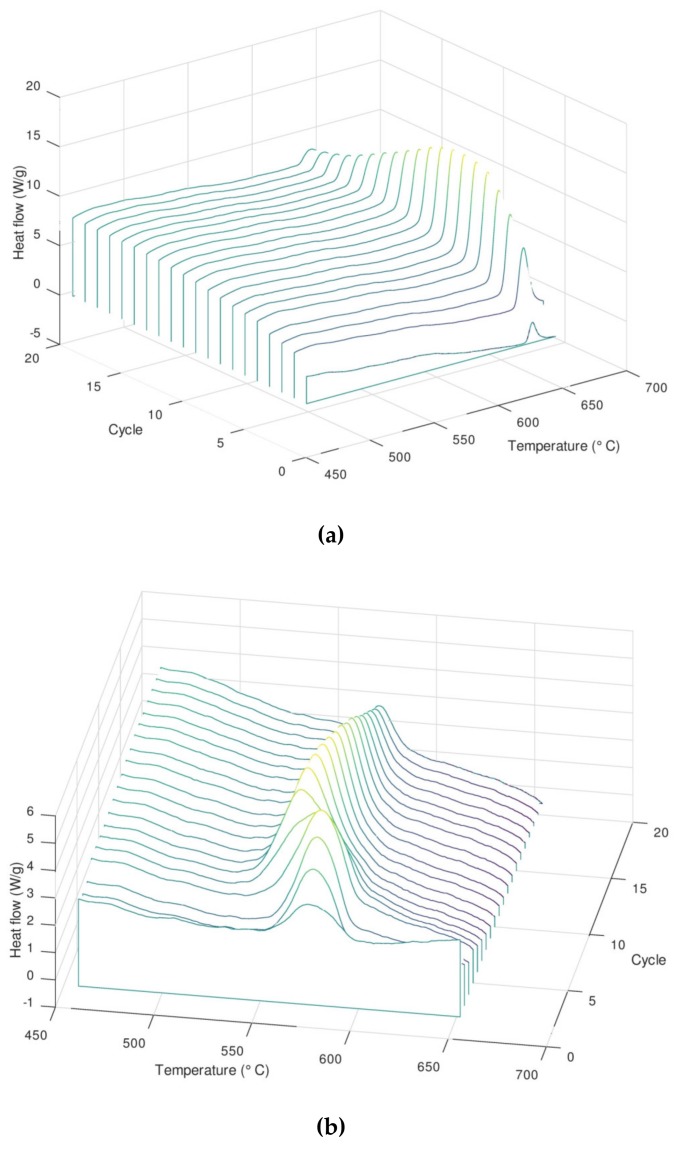
Cycling results of the sample Mn_75_-Ni_25_ synthesized by the two-step synthesis. (**a**) Heating steps, (**b**) cooling steps.

The reaction is reversible with a progressive increase of the enthalpy upon cycling. It is noteworthy that the value of the enthalpy for this system cannot be given precisely due to the very high reactivity of the material upon heating, leading to oxidation even when controlled atmosphere (level of oxygen below 0.1 ppm) or vacuum is applied. The energy obtained experimentally is considerably lower than the predicted one (around 10 J/g instead of 300 J/g). This behavior is amplified and clearly visible by performing cycling experiments in the DSC apparatus (up to 20 cycles), as shown in Figure 6. 

The cycling results show the progressive increase of the enthalpy of the reaction up to a certain limit, when the enthalpy starts to decrease to very low values (the DSC peak almost disappears). During the cooling process, a partial displacement of the peak during the first cycles can also be observed, reaching a stationary regime after eight cycles. In these experiments, two competitive effects are occurring at the same time, the progressive oxidation of the sample, which causes the increase of the inactive phase in the mixture (no contribution to the reaction heat), and the increase of the reaction enthalpy due to the intrinsic behavior of the mixture (still under investigation).

**Figure 7:**
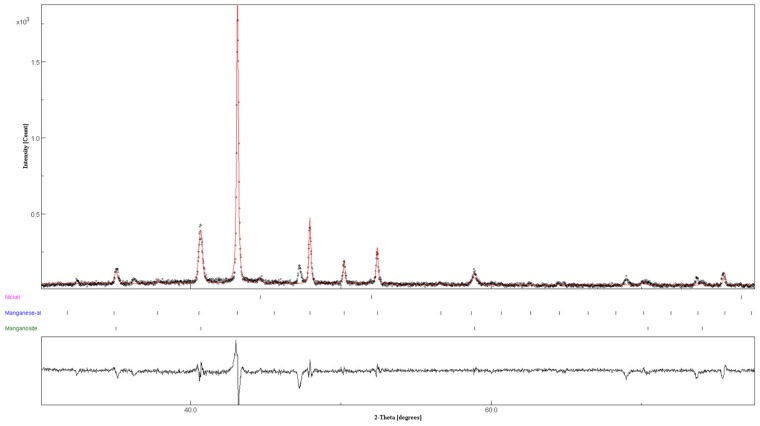
X-ray diffraction results of Mn_75_-Ni_25_ sample (two-step synthesis) after DSC experiment.

The X-ray diffraction analysis after the cycling experiment (20 cycles) did not allow correlation of the final structure with the reactivity observed. Only in the case of one of the samples cycled three times was it possible to obtain an XRD diffractogram (reported in Figure 7) in which the formation of a new unknown phase was detected (diffraction peaks at 2θ angular range of 36.2°, 47.2° and 68.9°). This phase could correspond to the intermetallic Mn_3_Ni predicted in the phase diagram; unfortunately, no crystallographic information is available for this phase, hindering its univocal determination.

**Figure 8:**
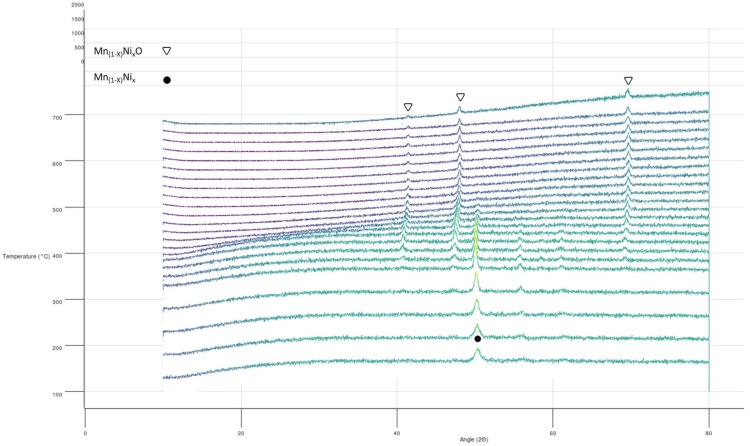
“In situ” X-ray diffraction measurements of Mn_75_-Ni_25_ sample (two-step synthesis).

To shed light on the reacting behavior observed and to determine the phenomena occurring upon heating several “in situ” XRD experiments were planned and carried out applying the same heating protocol used in the DSC experiments. The goal was to correlate the transitions observed in the DSC with the corresponding structural modification in the mixture. Unfortunately, none of the attempts made in order to obtain these measurements were successful, due to the extremely high reactivity of the mixture in the presence of traces of oxygen. Different trials were carried out under vacuum, under overpressure of N_2_ and in dynamic atmosphere (continuous vacuum and N_2_ flux). In all cases, the complete oxidation of the Mn_75_-Ni_25_ mixture was observed with the formation of the mixed oxide Mn_1−x_Ni_x_O (see Figure 8). It is noteworthy that, for the composition studied, the formation of the two solid solutions Mn_1−x_Ni_x_O and Ni_1−x_Mn_x_O should be detected, while only one phase was detected [21]. 

The same problems of oxidation were encountered when trying to perform measurements to test the thermophysical properties of Mn_75_-Ni_25_ upon heating (for examples in the case of LFA measurements), not making it possible to obtain further experimental results.

## 4. Conclusions and Perspectives

In this paper, solid-state reactions are proposed as possible candidates for thermal energy storage applications at high temperature. This study allowed the selection of several reactions with theoretical energy density above 100 kWh/m^3^. Two Mn-Ni compositions (Mn_75_-Ni_25_ and Mn_52_-Ni_48_) were chosen for the experimental study. The reaction was activated by thermal treatment after the preliminary preparation of nanostructured powders by ball milling techniques. The behavior of this system revealed considerably more complex than expected. More and more questions arose with the proceeding of the experimental investigation. For example, i) it is well known that mechanical milling is a powerful technique to increase the solubility limit in binary metallic systems; however, the solubilization of considerably high amount of nickel in the manganese net was accompanied by only a slight variation of the cell parameters. ii) A high degree of solubilization of Ni into Mn in the case of one-step synthesis where the two elements were milled at high milling intensity for up to 4 h was expected, while the results show an opposite behavior, with a higher solubilization degree being reached in the mixture prepared by two-step synthesis (the two elements milled together only for 15 min in mild conditions). iii) The direct milling of the two elements (one-step synthesis) led to a total absence of reactivity, even if similar degrees of nanocrystallization and homogenization were achieved, compared to the two-step synthesis. In addition, finally, what type of reacting event is connected to the reversible and progressively increasing peak observed in the DSC analysis? We are now pursuing different strategies to understand all of the behavior observed. The work is more focused on the material science point of view to explain the peculiar reactivity observed. Regarding the performance of this material for thermal energy storage applications, two main aspects can be considered. On one hand, the discrepancies between the theoretical reaction enthalpy and the experimental one are probably due to the progressive oxidation of the sample during thermal cycling. In this respect, it is hard to be definitive with regard to the performance as thermal energy storage material up to when it will be possible to test the material avoiding its oxidation. On the other hand, a material so reactive is not suitable for thermal energy storage applications because of the extremely controlled experimental conditions needed to avoid its degradation. This could be an important technological constraint leading, most probably, to investment costs that are too high for large-scale applications.

## Figures and Tables

**Table 1:** Results of the selection process.

System	Temperature °C	ΔHr J/mol	Volumetric Energy Density [kWh/m3]	Volumetric Energy Density (ΔT = 100 K)[kWh/m3]
Mn/Ni (52/48)	251	4281	166	281
Cu/Sn (84/16)	505	1505	48	98
Mn/Zn (68/32)	529	1407	48	149
Mn/Ni (25/75)	532	3746	171	331
Mn/Ni (75/25)	566	16774	583	707
Li_2_SO_4_/Na_2_SO_4_ (50/50)	515	40080	217	319
Al/Ni (38/62)	698	1504	52	176
Cu/Sn (75/25)	676	3124	84	465 (ΔT = 58 K)
Fe/Sn (60/40)	612	4011	97.7	176
Mn/Ni (41/59)	607	2784	117	265
Al/Mn (45/55)	842	6708	218	342
Fe/Si (30/70)	960	4996	165	263
Fe/Si (67/33)	962	3824	121	226

**Table 2:** Results of the Rietveld analysis for Mn_75_-Ni_25_ and Mn_52_-Ni_48_.

4 h Ball Milling	a (nm)	Error (nm)	<d> (nm)	Error (nm)	ε strain	Error
Mn (4 h)	0.89088	±4.1 × 10^−5^	19.3	±0.20	3.7 × 10^−3^	±9.2 × 10^−5^
Mn (Mn_75_-Ni_25_ One-step synthesis)	0.88802	±7.7 × 10^−5^	14.5	±0.27	1.8 × 10^−3^	±2.5 × 10^−4^
Mn (Mn_75_-Ni_25_ Two-step synthesis)	0.89107	±4.4 × 10^−5^	16.6	±0.16	2.6 × 10^−3^	±8.0 × 10^−5^
Mn (Mn_52_-Ni_48_ One-step synthesis)	0.88879	±9.7 × 10^−5^	14.4	±0.33	1.4 × 10^−3^	±4.4 × 10^−4^
Mn (Mn_52_-Ni_48_ Two-step synthesis)	0.89039	±9.7 × 10^−6^	17.2	±0.22	3.7 × 10^−3^	±6.6 × 10^−5^
